# Potentially traumatic life events and mental health conditions. Identifying the role of resilience in a cross-sectional study from Northwestern Germany

**DOI:** 10.3389/ijph.2026.1609376

**Published:** 2026-05-12

**Authors:** Felix Sisenop, Sarah Arndt, Kim-Julian Behr, Pallavi Chatarajupalli, Ingo Schäfer, Jutta Lindert

**Affiliations:** 1 Department of Social Work and Health, University of Applied Sciences Emden/Leer, Emden, Germany; 2 Department of Psychiatry and Psychotherapy, Centre for Interdisciplinary Addiction Research (ZIS), Universitatsklinikum Hamburg-Eppendorf, Hamburg, Germany

**Keywords:** cross-sectional, life events, mental health, resilience, rural population

## Abstract

**Objectives:**

Potentially traumatic life events (PTEs) are associated with increased risk of depression. Yet there is limited evidence on the association between quantity and type of PTEs and mental health in rural areas. This study examines the relationship between PTEs, resilience and mental health conditions (depression, anxiety) in Northwestern Germany.

**Methods:**

Cross-sectional population-based study of adults (n = 354) assessing sociodemographic factors, resilience (BRS), depression (PHQ-9), PTEs (LEC-5). Clusters of PTEs were identified on a conceptual basis. Linear regression analysis investigated association between PTEs and mental health. Resilience as a moderator was tested.

**Results:**

Depression was reported by 16.1% (n = 57), anxiety by 11.9% (n = 42) of the participants. PTE clusters identified were accidental/injury (n = 300, 84.7%), loss/life-threatening (n = 299, 84.5%), victimization (n = 218, 61.6%), and war/conflict-related events (n = 88, 24.9%). War/conflict-related events were linked with higher depression (B = 1.330, 95% CI: 0.013, 2.647). Resilience moderated the association between PTEs and depression.

**Conclusion:**

Cumulative and type of PTEs (especially war/conflict related) were associated with worse mental health. Resilience moderated associations with depression at lower resilience levels.

## Introduction

Mental health conditions (MHCs) such as depression and anxiety and their relationship with resilience are underexplored in rural populations. Resilience is broadly defined as the ability to adapt and recover [1, 2]. Depression and anxiety are among the most prevalent mental health conditions globally and within the European Region. In 2021, the 12-month prevalence rates were 4.3% for depression, and 5.4% for anxiety, contributing to 605.2 Disability-adjusted life years (DALYs) per 100,000 people, and 415.4 DALYs respectively [3, 4]. In Germany, 12-month prevalence estimates are even higher with estimates between 6% and 10% for depression, and 6.1% and 7.6% for anxiety [3, 5, 6]. In relation to potentially traumatic life events (PTEs) resilience is defined as “the ability of adults […] who are exposed to an isolated and potentially highly disruptive event […] to maintain relatively stable, healthy levels of psychological and physical functioning” [7]. Similarly, different approaches have been used to measure resilience: either by identifying the absence of MHCs such as depression, anxiety and PTSD, or by using resilience scales such as the Brief Resilience Scale [8] and the Connor-Davidson Resilience Scale [9]. Studies suggest that both mental health and resilience are influenced by exposure to PTEs [10–13]. Previous research findings show that resilience can mitigate the negative effects of PTEs on mental health [14–16].

Life events range from non-traumatic stressful events (e.g., interpersonal conflicts, job loss) to potentially traumatic life events such as violence, and war-related events [17–22].

However, the nature of the association between PTEs and MHCs is unclear and may depend on the type, number and context of PTEs. Some studies have proposed clusters of PTEs [23, 24]. Six clusters were identified in the World Mental Health Survey including collective violence, caused/witnessed bodily harm, interpersonal violence, intimate partner/sexual violence, accident/injuries using exploratory factor analysis [25]. Other studies have suggested different categories ranging between three (accidental/injury, victimization, predominant death threat) [24], four (assaultive violence, other injuries or shocking experiences, learning of traumatic events to close friends or relatives, sudden unexpected death of a close friend or relative) [26], and six clusters (physical assault/others, accidents/injury, natural disaster/witnessing death, sexual abuse, criminal assault, man-made disaster) [27]. Additionally, type of PTEs can be dichotomized, as intentional (e.g., direct assaults with a weapon) and unintentional (e.g., bereavement due to an accident) [28].

Mental health research has predominantly focused on urban regions due to both methodological convenience and specific content focus, leaving a critical gap in our understanding of rural populations. Most research focusses on urban regions. Urban and rural regions differ in terms of access to health services, infrastructure (access to job opportunities), and age distribution of the population [29, 30]. Access to healthcare structures in rural areas is worse than in urban areas [31–33]. There are fewer doctors, psychosocial support services, and psychotherapists and distances are longer [31]. This is especially important for people in vulnerable situations [34, 35]. Reduced access to healthcare and mental healthcare has negative effects on mental health outcomes in the population [36, 37]. In line with the differences between urban and rural regions, research in rural regions of Germany is neglected. In Germany, substantial regional disparities exist in the provision of mental healthcare [32]. Rural regions are characterized by a lower density of psychotherapists and psychiatrists compared to urban centers, leading to longer waiting times and travel distances [31–33]. Consequently, the institutionalized safety net available in cities is often thinner in rural areas, potentially increasing the reliance on individual resources like resilience to cope with PTEs. Germany’s mean age was higher in rural (45.76 years) than in urban (43.58 years) areas in 2023 [10]. The rural-urban disparity was also evident in the data on GDP *per capita* (37.55 vs. 50.83, data from 2022) and hospital beds per 1,000 people (5.26 vs. 6.02, data from 2022) [38]. The population-weighted mean distance to the next General Practitioner (GP) was more than double in rural (1.5 km) than in urban (0.7 km) areas in 2023 [38]. The study area is representative of rural transformation, characterized by an aging population and the emigration of younger individuals [39]. This exacerbates existing issues regarding access to healthcare and mental healthcare. We do not know yet, but it might be that due to the factors mentioned above there might be differences. Future analyses should compare urban and rural areas to determine whether differences in mental health outcomes and risk and protective factors for mental health vary based on level of rurality. To address this gap, the present study aims to investigate the association between exposure to PTEs MHCs, and the role of resilience in a rural population. Specifically, this study aims to 1) describe the rates of MHCs (anxiety and depression), levels of resilience, and exposure to PTEs in a sample from rural Germany, 2) identify clusters of PTEs, 3) investigate the associations of PTE exposure (cumulative number and type) with depression and anxiety, and 4) examine whether resilience moderates the association between PTE exposure and these MHCs.

## Methods

We report on a cross-sectional study conducted in Northwestern Germany (n = 503) (data collection 12/2022-10/2023). We conducted *a priori* power analysis for regression model 4, which had the highest number of predictors (n = 15), using G*Power 3.1. Assuming a small-to-moderate effect size, the required total sample size was 314. A total of n = 2,119 individuals were invited or registered themselves (n = 1,614) via postal invitations from municipal population registers for adults aged 60 years and over, and n = 505 via self-registration through community channels. We compared our study sample with routine statistics for the region (see Supplementary Table S1). We identified the following statistically significant differences between the general population and study participants: Our sample was older, comprised a higher proportion of women, and had higher educational levels than the general population (see Supplementary Table S1). Therefore, our sample does not claim representativeness.

### Sample and sample size calculation

Residents of Northwestern Germany aged ≥18 years who could provide informed consent were eligible. We used a dual-frame approach: 1) a stratified random sample of residents aged ≥60 years from municipal registers (stratified by municipality size, invitation by mail) and 2) a community-based convenience recruitment (all ages ≥18) via local channels (newspapers, social media, mailing lists). Surveys were primarily completed online (LimeSurvey), older adults were additionally offered postal paper-pencil questionnaires. Full sampling and recruitment details are reported elsewhere [40].

### Outcomes

We assessed mental health conditions, specifically depression and anxiety.

Depression was measured using the German version of the Patient Health Questionnaire-9 (PHQ-9) [41]. The PHQ-9 is a nine-item scale. Items are rated on a four-point Likert scale (“not at all,” “several days,” “more than half the days,” “nearly every day”), reflecting symptoms frequency over the past 2 weeks. A cut-off score of ≥10 was used to identify potential cases of depression, consistent with existent recommendations for the German population [41, 42].

Anxiety was assessed with the German version of the Generalized Anxiety Disorder 7 scale (GAD-7) [43]. The GAD-7 measures anxiety disorder with a seven-item scale rated on a four-point Likert scale (“not at all,” “several days,” “more than half the days,” “nearly every day”). A cut-off score of 10 was used to identify clinically relevant levels of anxiety [43]. It should be noted that scoring above the cut-off scores for the PHQ-9 and GAD-7 does not imply a clinical diagnosis.

The Cronbach’s α in our study sample indicated good internal consistency: PHQ-9: α = 0.858; GAD-7: α = 0.889.

### Influencing factors

Exposure to PTEs was assessed with an adapted version of the Life Events Checklist for DSM-5 (LEC-5) [44]. The LEC-5 is a widely used instrument to assess PTEs and provides a list of 16 PTEs with six response options (“happened to me,” “witnessed it,” “learned about it,” “part of my job,” “not sure,” “does not apply”). In line with other studies we defined exposure to a life event if one of the first four response options (“happened to me,” “witnessed it,” “learned about it,” “part of my job”) was selected [24]. Consistent with prior work, we chose this broader operationalization to capture exposures relevant for mental health in community samples [24]. Unlike the original format, which allows multiple responses for an item, our adaptation used single item response options for each life event [24, 45–47]. Exposure to PTEs was dichotomized to capture the exposure to PTEs in line with the PTSD DSM-5 Criterion A [48].

### Moderating factors

Resilience was measured using the German version of the Brief Resilience Scale (BRS) [8, 49], a six-item scale assessing resilience with a five-point Likert scale (“strongly disagree,” “disagree,” “neutral,” “agree,” “strongly agree,”). Computed scores below 3 are considered low resilience, between 3 and 4.3 normal resilience and above 4.3 high resilience [8]. Cronbach’s α of the BRS was α = 0.766 in our study sample.

### Potential covariates

We assessed several sociodemographic factors including age (continuous variable), gender (male, female, non-binary), marital status (single, in a partnership, married/registered civil partnership, married/registered civil partnership and living separately, divorced, widowed recoded to in a partnership, not in a partnership), highest achieved education level (elementary school, secondary school, entrance qualification for universities of applied sciences or equivalent, A-levels, undergraduate degree, postgraduate studies), employment status (employed, member of armed forces, school student, university student, pensioner, living from income from capital assets/renting/leasing, housewife/-man, not employed), and equivalized income in Euro (six original categories: <1,000; 1,000 - <2,000€; 2,000 - <3,000; 3,000 - <4,000; 4,000 - <5,000; ≥5,000) which was recoded for analysis into three categories <2,000, 2,000 – <4,000, ≥4,000).

### Statistical analysis

First, descriptive statistics were used to summarize sociodemographic factors, outcome variables, and the number and type of PTEs, using Pearson chi-square test for categorial variables and independent samples t-test and ANOVA for continuous variables. To group PTEs into meaningful categories, we relied primarily on a theoretical and conceptual approach guided by existing trauma literature [24]. We conducted a principal component analysis (PCA) with varimax rotation merely as an initial exploratory step to observe broad data patterns. The final assignment of events to four distinct clusters (victimization, accidental/injury, loss/life-threatening, and war/conflict-related) was based on content validity. The results of the exploratory PCA are displayed in the Supplementary Table S4. Subsequently, we conducted Linear Regression Analysis to estimate associations between PTEs and mental health outcomes, specifically depression and anxiety. We analyzed depression and anxiety and identified right-skewness. Despite this limitation, given the sample size (n = 354), we used linear regression models based on the Central Limit Theorem [50]. Potential heteroscedasticity in the linear regression models and moderation analysis was addressed by using the HC3 for heteroscedasticity-consistent standard error estimator. We applied a stepwise modelling approach: Model 1 included only sociodemographic variables, Model 2 assessed the effect of type of PTEs (accidental, loss, intentional, violence event), model 3 examined the impact of frequency of PTEs (0–1, 2–4, >4), model 4 combined both types of PTEs and sociodemographic variables to assess adjusted associations with the outcomes depression and anxiety. To explore potential mechanisms, we conducted moderation analysis using PROCESS v4.2 macro for SPSS 29. In the moderation model, resilience was included as a moderator between life event exposure and depression. Moderation analyses was exploratory. Analyses were not weighted and conducted for the total analytical sample.

## Results

### Participants

N = 505 people registered to participate online, additionally, n = 1,614 people were randomly selected to participate and contacted via mail letter. N = 555 people started the survey (response rate: 26.2%). Of these, n = 381 (68.7%) provided complete data on core variables (sociodemographics, PTEs, depression, anxiety, resilience). According to our non-responder analysis those who answered the last survey item had a mean age of 51.91 years (SD = 18.01, range: 19–89), n = 268 (67.7%) female and n = 128 (67.3%) male. The participants who did not complete the survey had a mean age of 44.53 (SD = 18.77, range: 18–88), n = 104 female (74.8%) and n = 35 (25.2%) male. The analytical sample included n = 354 participants living in the study area who provided complete responses to sociodemographic variables, outcome measures, and exposure to PTEs. A comparison of the analytical sample and the population in the research area regarding sociodemographic factors is available in Supplementary Table S1. We used the rurality definition of the German Federal Institute for Research on Building, Urban affairs and Spatial Development (BBSR) to differentiate between rural and urban areas [51]. The BBSR classifies districts based on the percentage of the population living in large and medium-sized cities, the population density of the district, and the population density excluding large and medium-sized cities [52]. The majority of the analytical sample lived in rural areas (n = 272, 76.8%).

### Sociodemographic characteristics of participants

The sociodemographic characteristics of the analytical sample, stratified by gender are presented in Table 1. The mean age of the participants was 52.32 years (SD = 17.72, range: 19–89). The majority of participants were female (66.4%), living in a partnership (76.0%), had an educational level of at least A-levels (70.9%), and equivalized income between 2,000–4,000€ (46.0%). Detailed sociodemographic characteristics are presented in Table 1.

**TABLE 1:** Sample characteristics by gender and number of life events (RISING study, Northwestern Germany, 2022–2023).

Characteristics	​	Gender					​	​	Number of life events					
​	Total		Male		Female		​	0–1		2–4		>4		
​	N	%	N	%	N	%	P^*^	N	%	N	%	N	%	P^*^
​	354	​	​	​	​	​	​	36	10.2	70	19.8	248	70.1	​
Gender	​	​	​	​	​	​	​	​	​	​	​	​	​	0.681
Male	119	33.6	-	-	-	-	​	13	36.1	25	35.7	81	32.7	​
Female	235	66.4	-	-	-	-	​	23	63.9	45	64.3	167	67.3	​
Age (years)	​	​	​	​	​	​	<0.001	​	​	​	​	​	​	0.128
Mean (SD, range)	52.32 (17.72,19–89)		56.88 (18.34, 21–89)		50.01 (16.97, 19–89)		<0.001	57.42 (19.09, 23–89)		57.42 (19.09, 23–89)		51.27 (17.25, 19–89)		0.075
18–29	63	17.8	17	14.3	46	19.6	​	5	13.9	13	18.6	45	18.1	​
30–39	33	9.3	10	8.4	23	9.8	​	2	5.6	5	7.1	26	10.5	​
40–49	43	12.1	8	6.7	35	14.9	​	4	11.1	9	12.9	30	12.1	​
50–59	66	18.6	14	11.8	52	22.1	​	6	16.7	11	15.7	49	19.8	​
60–69	85	24.0	38	31.9	47	20.0	​	6	16.7	14	20.0	65	26.2	​
70+	64	18.1	32	26.9	32	13.6	​	13	36.1	18	25.7	33	13.3	​
Marital status	​	​	​	​	​	​	0.210	​	​	​	​	​	​	0.176
No partnership	85	24.0	25	21.0	60	25.5	​	12	33.3	16	22.9	57	23.0	​
In partnership	269	76.0	94	79.0	175	74.5	​	24	66.7	54	77.1	191	77.0	​
Education	​	​	​	​	​	​	0.490	​	​	​	​	​	​	0.100
Below A-levels	103	29.1	34	28.6	69	29.4	​	15	41.7	18	25.7	70	28.2	​
At least A-levels	251	70.9	85	71.4	166	70.6	​	21	58.3	52	74.3	178	71.8	​
Working status	​	​	​	​	​	​	0.057	​	​	​	​	​	​	0.006
Not working	174	49.2	53	44.5	108	46.0	​	25	69.4	37	52.9	112	45.2	​
Working	180	50.8	66	55.5	127	54.0	​	11	30.6	33	47.1	136	54.8	​
Equivalized income	​	​	​	​	​	​	0.022	​	​	​	​	​	​	0.022
<2.000	149	42.1	45	37.8	104	44.3	​	13	36.1	29	41.4	107	43.1	​
2.000-<4.000	163	46.0	52	43.7	111	47.2	​	19	52.8	36	51.4	108	43.5	​
≥4.000	42	11.9	22	18.5	20	8.5	​	4	11.1	5	7.1	33	13.3	​
Depression (PHQ-9)	​	​	​	​	​	​	0.223	​	​	​	​	​	​	0.179
Mean (SD, range)	5.37 (4.73, 0–26)		4.56 (4.30, 0–23)		5.78 (4.88, 0–26)		0.008	3.53 (3.31, 0–14)		4.89 (4.05, 0–18)		5.78 (5.01, 0–26)		0.017
Yes	57	16.1	16	13.4	41	17.4	​	2	5.6	6	8.6	49	19.8	​
No	297	83.9	103	86.6	194	82.6	​	34	94.4	64	91.4	199	80.2	​
Anxiety (GAD-7)	​	​	​	​	​	​	0.009	​	​	​	​	​	​	0.835
Mean (SD, range)	3.98 (4.01, 0–21)		2.93 (3.43, 0–19)		4.51 (4.19, 0–21)		<0.001	2.53 (3.38, 0–15)		3.81 (3.96, 0–19)		4.23 (4.08, 0–21)		0.054
Yes	42	11.9	7	5.9	35	14.9	​	2	5.6	7	10.0	33	13.3	​
No	312	88.1	112	94.1	200	85.1	​	34	94.4	63	90.0	215	86.7	​
Resilience (BRS)	​	​	​	​	​	​	<0.001	​	​	​	​	​	​	0.396
Mean (SD, range)	20.65 (4.53, 6–30)		22.04 (4.44, 12–30)		19.94 (4–42, 6–30)		<0.001	20.44 (3.43, 16–30)		20.64 (4.05, 11–30)		20.68 (4.81, 6–30)		0.960
Low resilience	80	22.6	13	10.9	67	28.5	​	6	16.7	13	18.6	61	24.6	​
Medium resilience	227	64.1	81	68.1	146	62.1	​	27	75.0	49	70.0	151	60.9	​
High resilience	47	13.3	25	21.0	22	9.4	​	3	8.3	8	11.4	36	14.5	​

*p-values refer to global tests across groups (Pearson’s Chi-square tests for categorical variables; independent t-tests or ANOVAs, for continuous variables).

### Depression and anxiety outcome factors

Depression rates were 16.1% (n = 57) with a mean score of M = 5.37 (SD = 4.73, range: 0–26). The depression rates in our sample were slightly higher among females (17.4%, n = 41) compared to males (13.4%, n = 16). Overall anxiety was 11.9% with a mean score of 3.98 (SD = 4.01, range: 0–21) and anxiety levels were higher in the female sample (14.9%, n = 35) than in the male sample (5.9%, n = 7). Overall resilience was M = 20.65 (SD = 4.53, range: 6–30) with low resilience reported by 22.6% (n = 80) of the participants. Low resilience was reported more frequently by female participants (28.5%, n = 67) than by male participants (10.9%, n = 13), while high resilience was reported less frequently (males: 21.0%, n = 25, females: 9.4%, n = 22, see Table 1). Higher age (50–59 years: B = −2.156, 95% CI:-3.881, −0.430; 60 y-69 y: B = −2.450, 95% CI: −3.988, −0.912, 70 + y: B = −4.304, 95% CI: −6.038, −2.570) was negatively associated with depression and anxiety (50–59 years: B = −1.709, 95% CI: −3.171, −0.248; 60 y-69 y: B = −1.914, 95% CI: −3.217, −0.611, 70 + y: B = −3.576, 95% CI: −5.045, −2.106) compared to the reference group (19–29 years). Female gender (ref.: male) was associated with higher levels of anxiety (B = 1.054, 95% CI: 0.187, 1.920) and lower levels of resilience (B = −1.811, 95% CI: −2.831, −0.790). Lower education (ref: at least A-levels) was associated with higher depression (B = 1.174, 95% CI: 0.101, 2.246) and anxiety scores (B = 1.034, 95% CI: 0.126, 1.943, see Table 1).

### Potentially traumatic life events

The majority of participants reported exposure to more than four PTEs (70.1%, see Table 2). Four types of PTEs were identified: victimization events, accidental/injury events, loss/life threatening events, and war/conflict-related events. The most frequently reported types of PTEs were accidental/injury events (84.7%) and experience of loss/life-threatening events (84.5%), followed by victimization events (61.6%) and war/conflict-related being the least reported (24.9%, see Table 2). The results of the exploratory PCA are displayed in the Supplementary Table S4.

**TABLE 2:** Life events by gender (RISING study, Northwestern Germany, 2022–2023).

Life events	​		Gender				
​	Total		Male		Female		
​	N	%	N	%	N	%	P^*^
Number of traumatic life events	​	​	​	​	​	​	0.844
0–1 (ref.)	36	10.2	13	10.9	23	9.8	​
2–4	70	19.8	25	21.0	45	19.1	​
>4	248	70.1	81	68.1	167	71.1	​
Type of traumatic life events							
Victimization							
Yes	218	61.6	70	58.8	148	63.0	0.448
No	136	38.4	49	41.2	87	37.0	​
Accidental/injury							
Yes	300	84.7	107	89.9	193	82.1	0.054
No	54	15.3	12	10.1	42	17.9	​
Loss/life-threatening							
Yes	299	84.5	98	82.4	201	85.5	0.435
No	55	15.5	21	17.6	34	14.5	​
War/conflict-related							
Yes	88	24.9	36	30.3	52	22.1	0.095
No	266	75.1	83	69.7	183	77.9	​

*p-values are expressed in chi-squared values.

### Potentially traumatic life events and mental health conditions

In the adjusted regression models, having experienced more than four PTEs was associated with depression (B = 1.685, 95% CI: 0.569, 2.748) and anxiety (B = 1.290, 95% CI: 0.197, 2.382). Analysis of clusters of PTEs showed that exposure to war/conflict-related events was associated with depression (B = 1.330; 95% CI: 0.013, 2.647) (see Table 3).

**TABLE 3:** Linear regression: Associations between potentially traumatic life events (frequency, type) and mental health conditions (depression, anxiety) (RISING study, Northwestern Germany, 2022–2023).

Variables	Depression					Anxiety				
​	B	SE	95% CI lower	95% CI upper	P-value	B	SE	95% CI lower	95% CI upper	P-value
Model 1										
Any life events										
0–1 (ref.)										
2–4	1.358	0.742	−0.101	2.817	0.068	1.287	0.743	−0.176	2.749	0.084
>4	2.250	0.644	0.985	3.516	<0.001	1.706	0.627	0.473	2.939	0.007
Model 2										
Victimization event										
No (ref.)										
Yes	1.404	0.523	0.375	2.432	0.014	1.259	0.467	0.340	2.178	0.007
Accidental/injury event										
No (ref.)										
Yes	−0.813	0.670	−2.130	0.504	0.286	−0.851	0.639	−2.107	0.405	0.184
Loss/life-threatening event										
No (ref.)										
Yes	1.652	0.691	0.293	3.012	0.017	1.235	0.627	0.002	2.469	0.050
War/conflict event										
No (ref.)										
Yes	1.094	0.688	−0.259	2.447	0.113	0.691	0.579	−0.447	1.829	0.233
Model 3*										
Any life events										
0–1 (ref.)										
2–4	0.958	0.662	−0.344	2.260	0.149	0.960	0.669	−0.357	2.276	0.153
>4	1.658	0.554	0.569	2.748	0.003	1.290	0.555	0.197	2.382	0.021
Model 4*										
Victimization event										
No (ref.)										
Yes	0.616	0.504	−0.374	1.607	0.222	0.643	0.458	−0.257	1.544	0.161
Accidental/injury event										
No (ref.)										
Yes	−0.434	0.672	−1.756	0.887	0.518	−0.317	0.632	−1.561	0.926	0.616
Loss/life-threatening event										
No (ref.)										
Yes	1.365	0.700	−0.012	2.741	0.052	0.858	0.605	−0.332	2.047	0.157
War/conflict-related event										
No (ref.)										
Yes	1.330	0.670	0.013	2.647	0.048	0.988	0.552	−0.099	2.075	0.075

* Adjusted for age, gender, marital status, highest education level, employment status, equivalized income.

### Moderation analyses

**FIGURE 1:**
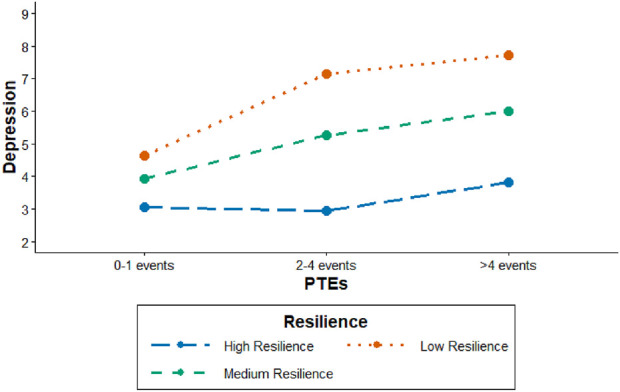
Moderator analysis: Simple slopes depicting the association between potentially traumatic life events and depression at varying levels of resilience as a moderator (RISING study, Northwestern Germany, 2022–2023).

Simple slope analysis revealed that relative to 0–1 events, a higher load of life events both 2-4 and >4 was associated with statistically significantly higher depression scores among participants with low and medium resilience. In contrast, no statistically significant association was observed for participants with high resilience (see Figure 1, Supplementary Table S5). These results indicate that the association between PTEs and depression varies by level of resilience, statistically significant for low and medium resilience and non-significant for high resilience.

### Sensitivity analyses

Given the well-established links between mental health and gender and education, we conducted sensitivity analysis by gender and education (below A-levels vs. at least A-levels), using the same moderation framework as in the main analysis. The results are presented as robustness checks, not confirmatory tests.

The interaction terms did not reach statistical significance in the gender-stratified models. Among male participants, high exposure to PTEs (>4 events) was statistically significantly associated with depression only in the low resilience group, but not in the medium or high resilience group. For female participants, exposure to PTEs was statistically significantly associated with higher depression scores in the low resilience group (at moderate and high exposure levels) and the medium resilience group (at high exposure levels). No statistically significant association was found in the high resilience group (see Supplementary Table S6).

In the low educational group simple slope analysis found a statistically significant moderation effect at the low and medium resilience level. No significant associations were observed at the high resilience level. In the higher education group resilience did not significantly moderate the association between PTEs and depression (see Supplementary Table S7).

## Discussion

The study aimed to 1) describe prevalence of MHCs, resilience levels and exposure to PTEs in a sample in rural Germany, 2) identify clusters of PTEs, and 3) investigate associations between number of PTEs and mental health outcomes including depression, anxiety and resilience and 4) test whether resilience moderates the association between PTEs and depression. The rates of depression (16.1%) in our sample exceed reported national averages in Germany (7.8%–11.7%) [5, 53]. Our findings are consistent with prior research showing that older age is associated with lower levels of depression and anxiety in Germany in contrast with European wide studies [54]. Female participants reported higher anxiety and depression and lower resilience scores, consistent with evidence from other studies [55–60].

Variations in rates of MHCs in our sample may be attributed to time-period effects, differences in sampling procedure, sample composition, and study locations. Notably, the sample analyzed here is mainly located in a rural region in Northwestern Germany, where access to healthcare and mental healthcare services is comparatively more limited than in urban areas. Studies have shown that limited access to healthcare services is negatively associated with mental health outcomes [61–63]. A substantial part of the population reported having experienced more than four PTEs which was associated with higher depression scores, aligning with previous findings on stress exposure and mental health [23, 64]. These findings are consistent with the allostatic load model [65], which suggests that chronic stress and repeated adversity can accumulate to a level that exceeds the body’s capacity to maintain psychological and physiological stability. While individuals may cope with isolated stressors, accumulation beyond a certain threshold can lead to dysregulation of stress-response systems, thereby increasing vulnerability to MHCs, such as depression [65].

Among the types of PTEs, war/conflict-related events were significantly associated with higher depression and anxiety scores. In contrast, more commonly reported events, such as accidents/injures or loss-related events, were not significantly associated with MHCs in this sample. These findings suggest that the extraordinary nature of certain events may play a crucial role in the relationship with MHCs. According to the Centrality of Events Theory, war/conflict-related events often function as pivotal life markers within a specific period during the life course [66]. Consistent with our findings, prior research also highlights the effect of loss events on depression and anxiety [67, 68]. Our findings highlight the importance of types of PTEs for MHCs, in line with other studies [24, 27, 69–73].

Moderation analysis indicated that resilience was associated with a weaker relationship between PTEs and depression. Specifically, a statistically significant association between PTEs and depression was observed among participating with low and medium resilience, and not statistically significant in the high resilience group. These findings suggest that resilience moderates the relationship between PTEs and depression, with the link not being observed in the high resilience group The moderation analysis did not find a moderating effect of resilience for the relationship between PTEs and anxiety. Long et al. [74] found that individuals who showed resilient trajectories with regard to depression following PTEs also demonstrated higher quality of life, greater perceived manageability, enhanced self-esteem and cognition, as well as more favorable physical health indicators such as body mass index. Future analyses should therefore examine whether resilience similarly moderates the impact of PTEs on a broader range of outcomes, indicating its potential as a transdiagnostic protective factor.

Our findings align with the stress-buffering model of resilience [75] and emphasize the need for targeted mental health policies and interventions tailored to the needs of people with lower resilience and lack of resilience factors. Highlighting the potential and importance of resilience interventions [76]. While the findings of this study relate specifically to Northwestern Germany, they could be highly relevant to other populations at acute risk of PTE due to violence, expulsion, and the collapse of social structures during natural or man-made disasters, such as war and flooding.

Although the interaction between resilience and PTEs was not statistically significant in male participants (*p* > 0.05), simple slope analyses were explored for descriptive purposes. These indicated that depression was associated with high PTE exposure in the low resilience group, whereas no such association was found at higher levels of resilience. However, given the cross-sectional nature of the data and the statistically non-significance of the interaction, these effects are no evidence for a buffering effect. Subgroup analysis stratified by education (low vs. high educational level) did not show significant interaction effects between resilience and PTEs. However, among participants with lower education, simple slope analysis indicated that the association between PTEs and depression remained significant at low and medium levels of resilience, consistent with patterns observed in the total sample. Among higher-education participants, simple slope analysis showed no significant associations across the resilience groups, except for high stressful PTEs exposure in the low resilience group. This finding should be interpreted cautiously, if at all, due to the non-significant interaction terms. This may indicate that, in participants with higher education, resilience plays a less central role in the association between PTEs and depression, possibly due to the presence of other protective factors (e.g., social support, coping style) not captured in our analysis.

In addition to the actual exposure to PTEs, the subjective appraisal of such events plays a crucial role in the development of MHCs. According to the Cognitive Appraisal Theory, individuals’ perceptions of the severity, controllability, or threat level of an event can significantly influence mental health outcomes [77]. Depending on the level of perceived valence, impact, threat or distress (e.g., perceived loss of vital resources) PTEs may contribute to higher rates of MHCs [78, 79]. Future studies should incorporate appraisal-sensitive instruments that assess perceived stress, threat, and controllability in order to better understand the mechanisms linking PTEs and MHCs.

This study is the first study in a rural region of Germany investigating PTEs, mental health conditions and mental health. To the best of our knowledge, there are no data on this study region. This study region is characterized by a dispersed settlement structure, demographic aging (increase of mean age of 39.4 in 2000 to 44.4 in 2024), and scarcity of access to (mental) healthcare. However, several limitations must be considered when interpreting the results of this study. First, the cross-sectional design of our study limits interpretability to associations, without providing information about directions of associations. For example, a current depressive episode may influence the self-reporting of resilience or bias the recall of PTEs. Therefore, longitudinal data are needed to analyze the temporal sequence of exposure, outcome and moderator as well as enable analysis of time-varying exposures. Due to the dual-frame sampling approach being used and difference and recruitment channels, our study does not pose representativeness (see Supplementary Table S1). Regarding sampling bias, non-random participation may have influenced the findings. Although the response rate is comparable to other population-based studies, it may introduce selection bias. The comparison with Census data of the greater study region revealed statistically significant differences between the sample and the general population regarding age and gender. Our sample was older and included more females compared to the general population. Also, people with current depressive episodes may be less likely to participate, possibly introducing bias as well. PTEs were assessed using a single response format to be in line with the PTSD DSM-5 Criterion A. For the analysis of cumulative trauma exposure, we analyzed exposure to PTE in regard to exposed vs. not exposed, thus not taking into account direct vs. indirect exposure. The PCA, used as a first step in clustering PTEs, is an exploratory approach backed by conceptually-driven clustering by the authors poses a methodological limitation. Resilience was assessed with the BRS, which may not fully capture resilience as a dynamic process over time when analyzed with cross-sectional data and does not include, e.g., external resilience factors. Future analyses of the RISING study will utilize longitudinal data to examine how changes in exposure and dynamic resilience factors affect mental health trajectories over time.
