# Supplementary material for: Potentially traumatic life events and mental health conditions. Identifying the role of resilience in a cross-sectional study from Northwestern Germany
Source: Int J Public Health. 2026 May 12;71:1609376. doi: 10.3389/ijph.2026.1609376 (PMC13201209; doi:10.3389/ijph.2026.1609376)
Supplement: Supplementary file 1 [file Supplementaryfile1.docx]

| **Variables** | **Sample** | | **Population (2022)** | |  |
| --- | --- | --- | --- | --- | --- |
|  | n | % | n | % | P^*^ |
| **Total** | 354 |  | 6583223 |  |  |
| **Gender** |  |  |  |  | <0.001 |
| Male | 119 | 33.6 | 3268433 | 49.7 |  |
| Female | 235 | 66.4 | 3407881 | 51.8 |  |
| **Age groups** |  |  |  |  | 0.001 |
| 18-29 | 63 | 17.8 | 1081611 | 16.4 |  |
| 30-39 | 33 | 9.3 | 881375 | 13.4 |  |
| 40-49 | 43 | 12.1 | 932274 | 14.2 |  |
| 50-59 | 66 | 18.6 | 1297300 | 19.7 |  |
| 60-69 | 85 | 24.0 | 1062540 | 16.1 |  |
| 70+ | 64 | 18.1 | 1328123 | 20.2 |  |
| **Marital status** |  |  |  |  | 0.283 |
| Not married/Not in a relationship | 85 | 24.0 | 1747000 | 26.54 |  |
| Married/In a relationship | 269 | 76.0 | 4836223 | 73.46 |  |
| **Household income**** |  |  |  |  | <0.001 |
| <2.000 | 79 | 22.3 | 1337000 | 20.31 |  |
| 2.000 - 4.000 | 158 | 44.6 | 1545000 | 23.47 |  |
| >4.000 | 117 | 33.1 | 1032000 | 15.68 |  |

**Table S1.** Comparison of the analytical sample with census data for Lower Saxony (RISING Study, Northwestern Germany, 2022–2023).

*p-values are expressed in chi-squared values, **For comparability reasons non-equivalised household net income was used.

**Table S2.** Correlation analysis (RISING Study, Northwestern Germany, 2022-2023).

|  | Gender | Age groups | Marital status | Highest obtained education | Working status | Household income net | Depression | Anxiety | Resilience | 2+ life events | Victimization event | Accidental/injury event | Loss/life-threatening event | War/conflict-related event |
| --- | --- | --- | --- | --- | --- | --- | --- | --- | --- | --- | --- | --- | --- | --- |
| 1 | 1.000 |  |  |  |  |  |  |  |  |  |  |  |  |  |
| 2 | -.198** | 1.000 |  |  |  |  |  |  |  |  |  |  |  |  |
| 3 | -0.050 | 0.020 | 1.000 |  |  |  |  |  |  |  |  |  |  |  |
| 4 | -0.008 | -.353** | 0.018 | 1.000 |  |  |  |  |  |  |  |  |  |  |
| 5 | -0.090 | .299** | -.188** | -.117* | 1.000 |  |  |  |  |  |  |  |  |  |
| 6 | -0.103 | .172** | .158** | .143** | -.317** | 1.000 |  |  |  |  |  |  |  |  |
| 7 | 0.051 | -.292** | -0.096 | 0.078 | 0.030 | -.204** | 1.000 |  |  |  |  |  |  |  |
| 8 | .132* | -.187** | -0.101 | .139** | 0.076 | -0.102 | .529** | 1.000 |  |  |  |  |  |  |
| 9 | -.234** | .200** | 0.004 | -0.048 | 0.027 | .105* | -.274** | -.333** | 1.000 |  |  |  |  |  |
| 10 | 0.018 | -0.104 | 0.073 | 0.093 | -.137* | -0.031 | 0.097 | 0.066 | -0.009 | 1.000 |  |  |  |  |
| 11 | 0.040 | -.205** | -0.036 | 0.082 | -0.060 | -0.070 | .172** | .146** | -0.081 | .388** | 1.000 |  |  |  |
| 12 | -0.102 | -0.026 | 0.037 | 0.057 | -.117* | 0.007 | 0.036 | 0.010 | 0.066 | .585** | .343** | 1.000 |  |  |
| 13 | 0.041 | -0.101 | 0.088 | 0.069 | -0.093 | -0.037 | .124* | 0.085 | -0.073 | .707** | .335** | .382** | 1.000 |  |
| 14 | -0.089 | -0.034 | 0.002 | 0.081 | -0.029 | 0.012 | .139** | .153** | -0.033 | .172** | .306** | .189** | .211** | 1.000 |

**Table S3.** Life events by resilience levels (RISING study, Northwestern Germany, 2022-2023).

|  | **Resilience** | | | | | | | | |
| --- | --- | --- | --- | --- | --- | --- | --- | --- | --- |
|  | Total | | Low (n=80) | | Medium  (n=227) | | | High (n=47) | |
| **Variables** | N | % | N | % | N | % | n | % | P^*^ |
| **Quantity of life events** |  |  |  |  |  |  |  |  | 0.396 |
| 0-1 (ref.) | 36 | 10.2 | 6 | 7.5 | 27 | 11.9 | 3 | 6.4 |  |
| 2-4 | 70 | 19.8 | 13 | 16.3 | 49 | 21.6 | 8 | 17.0 |  |
| >4 | 248 | 70.1 | 61 | 76.3 | 151 | 66.5 | 36 | 76.6 |  |
| **Type of life events** |  |  |  |  |  |  |  |  |  |
| Victimization |  |  |  |  |  |  |  |  | 0.019 |
| No | 136 | 38.4 | 21 | 26.3 | 99 | 43.6 | 16 | 34.0 |  |
| Yes | 218 | 61.6 | 59 | 73.8 | 128 | 56.4 | 31 | 66.0 |  |
| Accidental/injury | |  |  |  |  |  |  |  | 0.462 |
| No | 54 | 15.3 | 15 | 18.8 | 34 | 15.0 | 5 | 10.6 |  |
| Yes | 300 | 84.7 | 65 | 81.3 | 193 | 85.0 | 42 | 89.4 |  |
| Loss/life-threatening | | |  |  |  |  |  |  | 0.045 |
| No | 55 | 15.5 | 6 | 7.5 | 43 | 18.9 | 6 | 12.8 |  |
| Yes | 299 | 84.5 | 74 | 92.5 | 184 | 81.1 | 41 | 87.2 |  |
| War/conflict-related | | |  |  |  |  |  |  | 0.626 |
| No | 266 | 75.1 | 57 | 71.3 | 174 | 76.7 | 35 | 74.5 |  |
| Yes | 88 | 24.9 | 23 | 28.7 | 53 | 23.3 | 12 | 25.5 |  |

*p-values are expressed in chi-squared values

**Table S4.** Rotated component matrix (Varimax) of the LEC-5 in our sample (RISING study, Northwestern Germany, 2022–2023).

| **Item** | **Factor** | | | |
| --- | --- | --- | --- | --- |
|  | Victimization | Accidental/Injury | Loss/life-threatening | War/Conflict-related |
| Natural disaster |  | 0.778 |  |  |
| Fire/explosion |  | 0.757 |  |  |
| Transportation accident |  | 0.693 |  |  |
| Serious accident at work/home/during recreational activity | 0.329 | 0.452 | 0.328 |  |
| Exposure to toxic substance | 0.517 |  |  |  |
| Physical assault | 0.704 |  |  |  |
| Assault with a weapon | 0.721 |  |  |  |
| Sexual assault | 0.757 |  |  |  |
| Other unwanted/uncomfortable sexual experience | 0.728 |  |  |  |
| Combat exposure to war | 0.510 |  |  | 0.566 |
| Forced captivity | 0.300 |  |  | 0.737 |
| Life-threatening illness/injury |  |  | 0.727 |  |
| Severe human suffering |  |  | 0.801 |  |
| Sudden, violent death | 0.326 | |  | 0.518 |
| Sudden, accidental death | 0.323 |  | 0.543 |  |
| Serious injury/harm/death you caused to someone else |  |  |  | 0.725 |

**Table S5.** Moderation analysis: Full regression model evaluating the association between potentially traumatic events and depression with resilience as a moderator (RISING study, Northwestern Germany, 2022–2023).

| **Variables** | **B** | **SE** | **95% CI Lower** | **95% CI Upper** | **P-Value** |
| --- | --- | --- | --- | --- | --- |
| ***Constant*** | 8.431 | 2.050 | 4.399 | 12.463 | <0.001 |
| **PTEs** |  |  |  |  |  |
| 0-1 (ref.) |  |  |  |  |  |
| 2-4 | 7.223 | 3.266 | 0.799 | 13.648 | 0.028 |
| >4 | 7.253 | 2.534 | 2.269 | 12.237 | 0.005 |
| **Resilience** | -0.174 | 0.083 | -0.337 | -0.012 | 0.036 |
| **Interaction effects** |  |  |  |  |  |
| PTEs (2-4) × Resilience | -0.294 | 0.147 | -0.583 | -0.004 | 0.047 |
| PTEs (>4) × Resilience | -0.259 | 0.110 | -0.476 | -0.042 | 0.019 |
| ***Covariates*** |  |  |  |  |  |
| **Age** |  |  |  |  |  |
| 18-29 |  |  |  |  |  |
| 30-39 | -0.690 | 1.196 | -3.042 | 1.662 | 0.564 |
| 40-49 | -1.072 | 0.985 | -3.010 | 0.866 | 0.277 |
| 50-59 | -1.699 | 0.878 | -3.426 | 0.029 | 0.054 |
| 60-69 | -2.354 | 0.802 | -3.932 | -0.776 | 0.004 |
| 70+ | -3.597 | 0.834 | -5.238 | -1.957 | <0.001 |
| **Gender** |  |  |  |  |  |
| Male (ref.) |  |  |  |  |  |
| Female | -0.280 | 0.491 | -1.246 | 0.686 | 0.569 |
| **Marital status** |  |  |  |  |  |
| No Partnership (ref.) |  |  |  |  |  |
| In Partnership | 1.139 | 0.615 | -0.071 | 2.349 | 0.065 |
| **Education** |  |  |  |  |  |
| Below A-levels (ref.) |  |  |  |  |  |
| At least A-levels | 0.701 | 0.535 | -0.352 | 1.754 | 0.191 |
| **Working status** |  |  |  |  |  |
| Not working (ref.) |  |  |  |  |  |
| Working | 0.391 | 0.697 | -0.979 | 1.761 | 0.575 |
| **Equivalized income** |  |  |  |  |  |
| <2,000 (ref.) |  |  |  |  |  |
| 2,000 - <4,000 | 0.452 | 0.699 | -0.923 | 1.827 | 0.518 |
| ≥4,000 | 0.077 | 0.640 | -1.181 | 1.340 | 0.904 |

**Table S6.** Moderation analysis: Full regression models stratified by gender evaluating the association between potentially traumatic events and depression with resilience as a moderator (RISING study, Northwestern Germany, 2022–2023).

|  | **Gender** | | | | | | | | | |
| --- | --- | --- | --- | --- | --- | --- | --- | --- | --- | --- |
|  | **Male** | | | | | **Female** | | | | |
| **Variables** | **B** | **SE** | **95% CI Lower** | **95% CI Upper** | **P** | **B** | **SE** | **95% CI Lower** | **95% CI Upper** | **P** |
| ***Constant*** | 7.023 | 9.785 | -12.383 | 26.428 | 0.475 | 9.292 | 2.429 | 4.505 | 14.080 | <0.001 |
| **PTEs** |  |  |  |  |  |  |  |  |  |  |
| 0-1 (ref.) | - | - | - | - | - | - | - | - | - | - |
| 2-4 | 6.096 | 11.488 | -16.688 | 28.880 | 0.597 | 8.253 | 3.952 | 0.465 | 16.042 | 0.038 |
| >4 | 11.223 | 10.009 | -8.627 | 31.073 | 0.265 | 6.561 | 3.071 | 0.510 | 12.613 | 0.034 |
| **Resilience** | -0.004 | 0.478 | -0.953 | 0.945 | 0.994 | -0.204 | 0.092 | -0.385 | -0.023 | 0.028 |
| **Interaction effects** |  |  |  |  |  |  |  |  |  |  |
| PTEs (2-4) × Resilience | -0.266 | 0.562 | -1.380 | 0.849 | 0.638 | -0.337 | 0.177 | -0.686 | 0.011 | 0.058 |
| PTEs (>4) × Resilience | -0.444 | 0.498 | -1.432 | 0.544 | 0.375 | -0.231 | 0.131 | -0.489 | 0.026 | 0.078 |
| ***Covariates*** |  |  |  |  |  |  |  |  |  |  |
| **Age** |  |  |  |  |  |  |  |  |  |  |
| 18-29 (ref.) |  |  |  |  |  |  |  |  |  |  |
| 30-39 | -0.377 | 2.174 | -4.688 | 3.934 | 0.863 | -0.752 | 1.525 | -3.757 | 2.254 | 0.623 |
| 40-49 | -1.283 | 2.641 | -6.521 | 3.955 | 0.628 | -0.874 | 1.170 | -3.181 | 1.433 | 0.456 |
| 50-59 | -1.988 | 1.733 | -5.425 | 1.448 | 0.254 | -1.419 | 1.069 | -3.526 | 0.688 | 0.186 |
| 60-69 | -1.921 | 1.284 | -4.468 | 0.625 | 0.138 | -2.598 | 1.087 | -4.740 | -0.456 | 0.018 |
| 70+ | -2.939 | 1.278 | -5.474 | -0.405 | 0.024 | -3.806 | 1.101 | -5.977 | -1.635 | 0.001 |
| **Marital status** |  |  |  |  |  |  |  |  |  |  |
| No Partnership (ref.) |  |  |  |  |  |  |  |  |  |  |
| In Partnership | -1.595 | 1.106 | -3.788 | 0.598 | 0.152 | -1.073 | 0.809 | -2.667 | 0.522 | 0.186 |
| **Education** |  |  |  |  |  |  |  |  |  |  |
| Below A-levels (ref.) |  |  |  |  |  |  |  |  |  |  |
| At least A-levels | -0.003 | 1.042 | -2.070 | 2.065 | 0.998 | 1.050 | 0.676 | -0.282 | 2.382 | 0.122 |
| **Working status** |  |  |  |  |  |  |  |  |  |  |
| Not working (ref.) |  |  |  |  |  |  |  |  |  |  |
| Working | -0.663 | 1.224 | -3.092 | 1.765 | 0.589 | 0.941 | 0.909 | -0.850 | 2.731 | 0.302 |
| **Equivalized income** |  |  |  |  |  |  |  |  |  |  |
| <2,000 (ref.) |  |  |  |  |  |  |  |  |  |  |
| 2,000 - <4,000 | 0.569 | 1.220 | -1.851 | 2.988 | 0.642 | 0.608 | 0.961 | -1.286 | 2.502 | 0.528 |
| ≥4,000 | -0.337 | 0.976 | -2.274 | 1.600 | 0.731 | 0.453 | 0.874 | -1.270 | 2.175 | 0.605 |

**Table S7.** Moderation analysis: Full regression models stratified by education evaluating the association between potentially traumatic events and depression with resilience as a moderator (RISING study, Northwestern Germany, 2022–2023).

|  | **Education** | | | | | | | | | |
| --- | --- | --- | --- | --- | --- | --- | --- | --- | --- | --- |
|  | **Below A-levels (n=103)** | | | | | **At least A-levels (n=251)** | | | | |
| **Variables** | **B** | **SE** | **95% CI Lower** | **95% CI Upper** | **P** | **B** | **SE** | **95% CI Lower** | **95% CI Upper** | **P** |
| ***Constant*** | 4.362 | 5.374 | -6.320 | 15.043 | 0.419 | 8.629 | 2.667 | 3.376 | 13.883 | 0.001 |
| **PTEs** |  |  |  |  |  |  |  |  |  |  |
| 0-1 (ref.) | - | - | - | - | - | - | - | - | - | - |
| 2-4 | 10.392 | 5.343 | -0.227 | 21.011 | 0.055 | 7.479 | 4.438 | -1.265 | 16.223 | 0.093 |
| >4 | 10.201 | 4.379 | 1.498 | 18.903 | 0.022 | 6.925 | 3.297 | 0.430 | 13.419 | 0.037 |
| **Resilience** | -0.081 | 0.158 | -0.395 | 0.234 | 0.612 | -0.157 | 0.123 | -0.399 | 0.085 | 0.203 |
| **Interaction effects** |  |  |  |  |  |  |  |  |  |  |
| PTEs (2-4) × Resilience | -0.350 | 0.233 | -0.812 | 0.113 | 0.137 | -0.345 | 0.209 | -0.758 | 0.067 | 0.101 |
| PTEs (>4) × Resilience | -0.360 | 0.196 | -0.750 | 0.031 | 0.071 | -0.275 | 0.153 | -0.577 | 0.026 | 0.073 |
| ***Covariates*** |  |  |  |  |  |  |  |  |  |  |
| **Age** |  |  |  |  |  |  |  |  |  |  |
| 18-29 (ref.) |  |  |  |  |  |  |  |  |  |  |
| 30-39 | 7.930 | 6.944 | -5.872 | 21.732 | 0.257 | -1.550 | 1.217 | -3.947 | 0.847 | 0.204 |
| 40-49 | 2.148 | 4.239 | -6.279 | 10.574 | 0.614 | -1.268 | 1.132 | -3.498 | 0.962 | 0.264 |
| 50-59 | 1.638 | 4.090 | -6.491 | 9.767 | 0.690 | -2.155 | 1.042 | -4.208 | -0.101 | 0.040 |
| 60-69 | 0.712 | 4.249 | -7.733 | 9.157 | 0.867 | -2.441 | 1.000 | -4.410 | -0.472 | 0.015 |
| 70+ | -0.557 | 4.316 | -9.136 | 8.021 | 0.898 | -3.697 | 0.936 | -5.541 | -1.854 | <0.001 |
| **Gender** |  |  |  |  |  |  |  |  |  |  |
| Male (ref.) |  |  |  |  |  |  |  |  |  |  |
| Female | -0.890 | 0.933 | -2.745 | 0.965 | 0.343 | 0.003 | 0.618 | -1.216 | 1.221 | 0.996 |
| **Marital status** |  |  |  |  |  |  |  |  |  |  |
| No Partnership (ref.) |  |  |  |  |  |  |  |  |  |  |
| In Partnership | 1.159 | 0.971 | -0.771 | 3.090 | 0.236 | 1.112 | 0.786 | -0.437 | 2.662 | 0.159 |
| **Working status** |  |  |  |  |  |  |  |  |  |  |
| Not working (ref.) |  |  |  |  |  |  |  |  |  |  |
| Working | -0.431 | 1.465 | -3.343 | 2.482 | 0.769 | 0.742 | 0.822 | -0.877 | 2.360 | 0.368 |
| **Equivalized income** |  |  |  |  |  |  |  |  |  |  |
| <2,000 (ref.) |  |  |  |  |  |  |  |  |  |  |
| 2,000 - <4,000 | -0.489 | 1.412 | -3.295 | 2.319 | 0.730 | 0.604 | 0.859 | -1.089 | 2.297 | 0.483 |
| ≥4,000 | -0.822 | 1.352 | -3.509 | 1.866 | 0.545 | 0.290 | 0.734 | -1.156 | 1.737 | 0.693 |
